# Anesthesia for cataract surgery: Recent trends

**DOI:** 10.4103/0974-620X.71881

**Published:** 2010

**Authors:** Rikin Shah

**Affiliations:** Department of Ophthalmology, Al Nahdha Hospital, Ruwi, Oman

The world has witnessed a significant evolution in surgical technique of cataract extraction in last few decades. After Ridley introduced the intraocular lens, the challenge was to reduce the size of incision. It was fulfilled by Kelman with the introduction of phacoemulsification and by Mazzocco with the introduction of foldable intraocular lens. Of course advances in phaco machines, phacotips, ophthalmic viscosurgical devices (OVD), etc. also have played a major role to reach today’s faster, more controlled, and less traumatic cataract surgery.

As incisional size of cataract extraction has reduced, anesthesia techniques have also advanced significantly. General anesthesia was introduced in mid-19th century. Koller and Knapp can be considered the pioneers of local anesthesia for cataract surgery. Koller introduced topical cocaine in 1884 while Knapp introduced retrobulbar anesthesia in 1884. In the beginning of 19^th^ century, orbicularis block was introduced by Van Lint, O’Beriens, and Alkinson. In last 25 years, local anesthesia techniques have progressed from posterior peribulbar to “no anesthesia” techniques.

Peribulbar and retrobulbar techniques are associated with a risk for complications such as globe perforation, damage to optic nerve, retrobulbar hemorrhage, and ocular muscle injury. Rarely, they can be life-threatening. Introduction of the sub-Tenon’s anesthesia technique reduced the risk of complications of peribulbar/retrobulbar anesthesia but the technique is still associated with a possibility of all the complications of peribulbar/retrobulbar techniques. Evolving surgical techniques have reduced the need for akinesia. In 1992, Fichman reintroduced topical anesthesia for cataract surgery. Topical anesthesia is used to block the afferent nerves of the corneal and the conjunctiva (long and short ciliary nerves, nasociliary, and lacrimal nerves). This technique eliminates the possible complications of injectable anesthesia. However, it does not eliminate pain sensitivity of the iris, the zonule, and the ciliary body. In 1992, Gills introduced intracameral technique of anesthesia with preservative free 1% lidocaine. In 1999, Koch-Assia introduced use of Xylocaine jelly for surface anesthesia. Today different agents are available in market for topical anesthesia like Procaine (1%/2%/10%), Proparacaine (0.5%), Oxybuprocaine (0.4%), Tetracaine (0.5%/1%), Bupivacaine (0.25%/0.5%), Etidocaine (1%), Lidocaine (0.5%/1%), Prilocaine (4%), and Ropicacaine (0.2%/1%). All these agents have different time of onset and duration of anesthesia. Topical and intracameral techniques are not absolutely safe as epithelial and endothelial toxicities are reported with them.

In 1998, Amar Agarwal introduced the technique of “no anesthesia” for cataract extraction. In this technique, no topical or intracameral drugs are used. Although without any side effects, the stress for the surgeon is increased. A question that arises is - cornea is supplied by a dense plexus of sensory nerves. Then how it is possible to do cataract surgery without any anesthesia? Possible explanations are: peripheral and superior cornea is less sensitive than central cornea, dark-brown eyed Indians, Chinese, and blacks have a corneal sensitivity that is four times less than blue-eyed Caucasians, people in developing countries like India are more exposed to ultraviolet rays which may result in a significant loss of corneal sensitivity. Due to increased stress on surgeon, the “no anesthesia” technique has not gained popularity in the western world.

In 1999, Gutierrez–Carmona modified “no anesthesia” technique and introduced cryoanalgesia for cataract surgery. In this technique, all solutions to be used during surgery are cooled to 4° C except povidone drops. Before surgery, an eye mask of cold gel is placed over the eye for 10 min. During the surgery, the eye is irrigated with cold balanced salt solution (BSS). All OVD used during surgery are cooled to 4° C. Although showed to be a safe technique for clear cornea phacoemulsification with acceptable level of pain, it is not suitable for all cataracts and all patients.

Is there any role for general anesthesia in modern cataract surgery? Of course yes—for pediatric cataracts and for some adult patients (i.e., mental retardation). The concept of providing different pharmacological agents for balanced anesthesia, i.e., for analgesia, muscle relaxant, abolishment of all reflexes including somatic reflexes, and somnolence has removed the use of very potent, toxic, and long-acting agents. The drugs used include: Opioids (μ receptor agonist): Fentanyl, Remifentanil, Alfentanil, Benzodiazepine (sedative/hypnotic): Midazolam, anesthetic gases: Isoflurane, Sevoflurane, and intravenous anesthetic agent Propofol (which in low dose can be used as sedative with topical anesthesia).

The million dollar question is: which anesthesia to select for cataract surgery? Only two persons can decide this—the patient, who is undergoing cataract surgery and the ophthalmic surgeon, who is going to operate. For the same patient, different surgeons may select different techniques of anesthesia. The skill and experience of surgeon, co-operation of patient, type of cataract, associated ocular co-morbidity like corneal opacity, pupillary dilatation, etc. are important factors while deciding upon the type of anesthesia.

Studies have showed different trends in different countries. A national postal survey was conducted in 2008 in the United Kingdom for choice of local anesthetic techniques. Sub-Tenon’s anesthesia was the local anesthetic technique of choice (47% compared to topical 33%, peribulbar 16%, retrobulbar 2%, and others 2%). Of sub-Tenon blocks, 28% were given by surgeons and 47% by the anesthetist. A similar survey done in Singapore in 2004 showed 92% cataract extraction were done by phacoemulsification technique. For phacoemulsification technique, the anesthetic technique of choice was peribulbar anesthesia (43%). A survey of members of the American Society of Cataract and Refractive Surgeons (ASCRS) in 2000 revealed an increase in the use of topical anesthesia among surgeons. In Oman, over the last few years, anesthesia for cataract surgery has shifted from general to local anesthesia.

